# A Root Specific Induction of Carotenoid Biosynthesis Contributes to ABA Production upon Salt Stress in Arabidopsis

**DOI:** 10.1371/journal.pone.0090765

**Published:** 2014-03-04

**Authors:** M. Águila Ruiz-Sola, Vicent Arbona, Aurelio Gómez-Cadenas, Manuel Rodríguez-Concepción, Antía Rodríguez-Villalón

**Affiliations:** 1 Centre for Research in Agricultural Genomics (CRAG) CSIC-IRTA-UAB-UB, Barcelona, Spain; 2 Departament de Ciències Agràries i del Medi Natural, Universitat Jaume I, Castelló de la Plana, Spain; University College Dublin, Ireland

## Abstract

Abscisic acid (ABA) is a hormone that plays a vital role in mediating abiotic stress responses in plants. Salt exposure induces the synthesis of ABA through the cleavage of carotenoid precursors (xanthophylls), which are found at very low levels in roots. Here we show that *de novo* ABA biosynthesis in salt-treated *Arabidopsis thaliana* roots involves an organ-specific induction of the carotenoid biosynthetic pathway. Upregulation of the genes encoding phytoene synthase (PSY) and other enzymes of the pathway producing ABA precursors was observed in roots but not in shoots after salt exposure. A pharmacological block of the carotenoid pathway substantially reduced ABA levels in stressed roots, confirming that an increase in carotenoid accumulation contributes to fuel hormone production after salt exposure. Treatment with exogenous ABA was also found to upregulate *PSY* expression only in roots, suggesting an organ-specific feedback regulation of the carotenoid pathway by ABA. Taken together, our results show that the presence of high concentrations of salt in the growth medium rapidly triggers a root-specific activation of the carotenoid pathway, probably to ensure a proper supply of ABA precursors required for a sustained production of the hormone.

## Introduction

**Figure 1:**
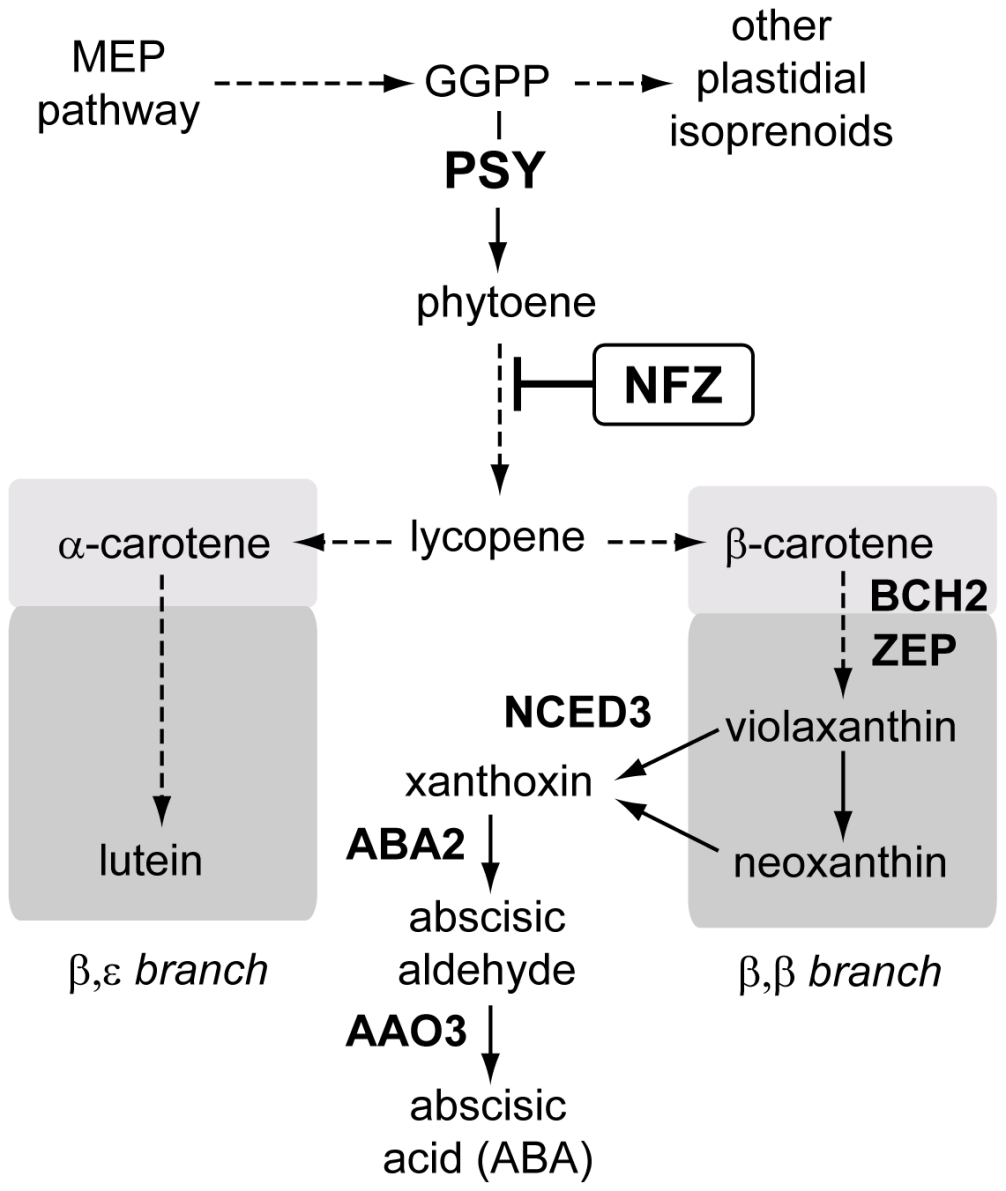
Carotenoid biosynthesis pathway. Enzymes are indicated in bold and correspond to the Arabidopsis genes *PSY* (At5g17230), *BCH2/CHY2* (At5g52570), *ZEP/ABA1/NPQ2* (At5g67030), *NCED3*/*SIS7* (At3g14440), *ABA2/GIN1/XD* (At1g52340), and *AAO3/ABAO* (At2g27150). The block of phytoene desaturation by norflurazon (NFZ) is indicated. Dashed arrows represent several steps. GGPP, geranylgeranyldiphosphate.

To cope with abiotic stress, including water deficit or salinity, plants have evolved various adaptative responses. The best understood are those mediated by the hormone abscisic acid (ABA). In particular, salt stress involves a massive accumulation of this hormone, which is generated through the cleavage of carotenoid precursors (xanthophylls) by specific dioxygenases [1], [2], [3], [4]. Among them, 9-cis-epoxycarotenoid dioxygenase 3 (NCED3) (Fig. 1) has an essential role in ABA production during salt stress [1], [3]. However, additional bottlenecks might exist, including the supply of upstream carotenoid precursors.

Carotenoids are isoprenoids synthesized by photosynthetic organisms and some non-photosynthetic bacteria and fungi. In plants, they are synthesized in plastids, where they play different functions. In chloroplasts of photosynthetic tissues, carotenoids contribute to light harvesting and photoprotection [5]. In some plant species, they also accumulate at high levels in chromoplasts of flowers and fruits, contributing to their colors [6], [7]. Carotenoids are also present, at much lower levels, in other non-photosynthetic plastids such as the etioplasts of dark-grown seedlings and the leucoplasts of roots [7].

The first specific reaction of the carotenoid pathway (Fig. 1) is the production of phytoene from two geranylgeranyldiphosphate molecules catalyzed by phytoene synthase (PSY). Uncoloredphytoene is then desaturated and isomerized to lycopene, a red carotenoid that can be further converted to α-carotene (β,ε-carotene) by introduction of β and ε rings or β-carotene (β,β-carotene) by introduction of two β rings. Hydroxylation of these carotenes generates β,ε-xanthophylls (lutein) and β,β-xanthophylls (violaxanthin, neoxanthin), respectively [8]. β-carotene and derived β,β-xanthophylls serve as precursors for important growth regulators, such as strigolactones and ABA, which regulate plant development and interaction with the environment [3], [4], [9]. In particular, ABA is produced from β,β-xanthophylls *via* xanthoxin by the activity of NCED3. A dehydrogenase/reductase encoded by the *ABA2* gene catalyses the conversion of xanthoxin to abscisic aldehyde, which is then oxidized into ABA by aldehyde oxidase 3 (AAO3) [3] (Fig. 1).

Unlike most plants, the *Arabidopsis thaliana* genome only contains a single gene (At5g17230) encoding PSY [8]. In plants with several PSY isoforms, at least one of them typically shows preferential expression in the root. This is the case of the maize and rice PSY3 [10], [11]. In agreement with a role of PSY in the production of ABA in the root, the expression of rice and maize *PSY3* genes is upregulated in roots upon saline, osmotic, or water stress [10], [11], [12]. The single Arabidopsis *PSY* gene is also expressed in the root [13], [14]. Overexpression of *PSY* results in higher carotenoid levels in Arabidopsis roots, with a preferential accumulation of β-carotene [14], suggesting that, similar to that observed in cotyledons of dark-grown seedlings [15], PSY activity might limit carotenoid biosynthesis rates in the root. However, it remains to be demonstrated whether physiological changes in the expression of the Arabidopsis *PSY* gene might influence root carotenoid or ABA levels.

Here we report that the salt-induced burst in ABA production involves an increase of carotenoids specifically in roots. We demonstrate that the upregulation of Arabidopsis *PSY* gene expression in stressed roots correlates with an increased production of β,β-xanthophylls, the substrates for *de novo* ABA biosynthesis. Furthermore, we show that ABA modulates this organ-specific response. Together, we provide evidence of a conserved root-specific feedback mechanism by which ABA regulates the biosynthesis of its own precursors after salt exposure.

## Results, Discussion, and Conclusions

### Carotenoid Accumulationis Enhancedin Salt-treated Roots but not in Shoots

**Figure 2:**
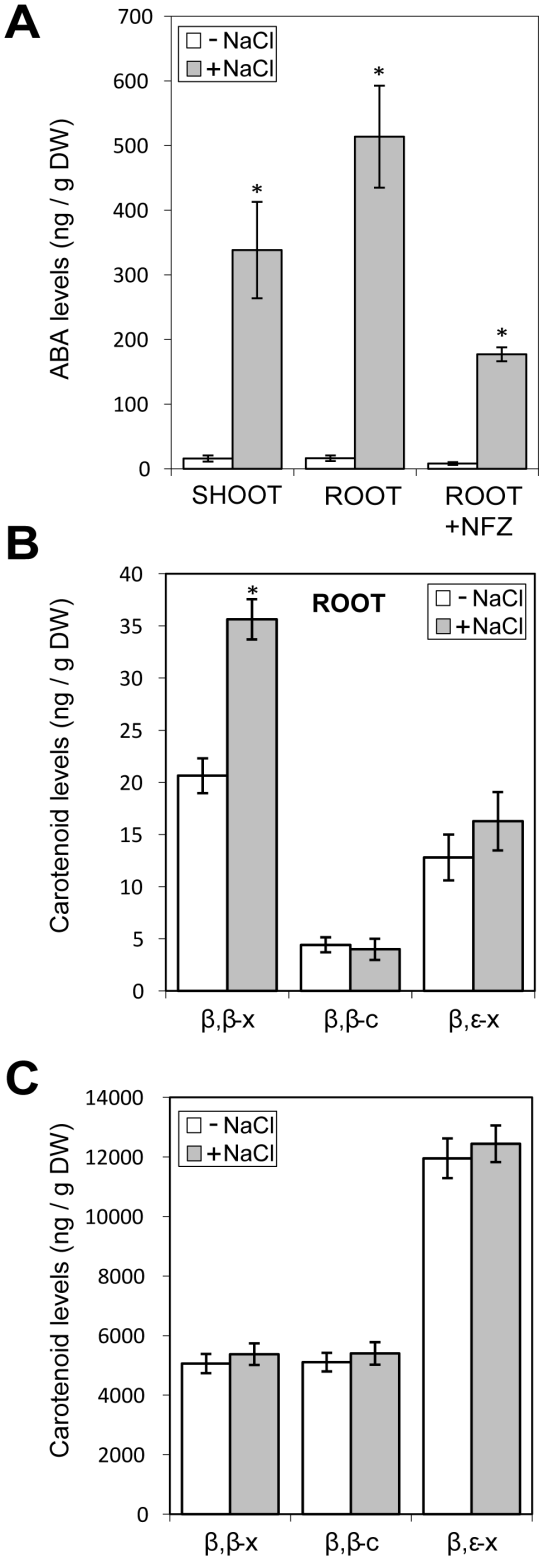
Effect of salt stress on ABA and carotenoid accumulation. (A) ABA quantification by LC-MS of root and shoot tissues from plants either treated (+) or not (−) with 200 mMNaCl for 5 h. The indicated samples were incubated with 20 µM norflurazon (NFZ) 48 h before and during the salt treatment. (B) Levels of β,βxanthophylls (β,β-x: neoxanthin and violaxanthin), β,β carotenes (β,β-c: β-carotene), and β,ε xanthophylls (β,ε-x: lutein) in root and shoot tissues separated from plants either treated (+) or not (−) with NaCl for 5 h. Data correspond to the mean and standard deviation of n = 3 independent samples. Asterisks mark statistically significant differences (p<0.01) relative to mock-treated controls.

It is well established that salt stress increases endogenous levels of ABA through the cleavage of β,β-xanthophylls [1], [3], [16] (Fig. 1). Arabidopsis roots have been suggested as a major source of ABA [3], [17], even if xanthophylls levels in this organ are scarce. It is therefore possible that, similar to that proposed in maize and rice roots [10], [11], an increase in the levels of these carotenoid-derived precursors might be required to sustain a salt-induced ABA production. To test this hypothesis, we decided to analyse carotenoid levels after exposing plants to salinity. Seeds were germinated on a mesh placed on top of solid MS medium in square plates that were incubated vertically. After growth for 2 weeks under cycles of 16 h light and 8 h dark (long day photoperiod, LD), the plants were transferred on the mesh to fresh medium either supplemented or not with 200 mM NaCl and shoot and root samples were collected separately after 5 h of treatment. As expected, analysis of ABA levels showed that salt exposure triggered a dramatic accumulation of this hormone both in root and aerial tissues (Fig. 2A). However, salt stress did not alter the carotenoid profile in shoot tissues but it increased the levels of xanthophylls in roots compared to mock-control samples (Fig. 2B). The differences relative to mock samples were only statistically significant (p<0.01, T-test) for xanthophylls of the β,β branch (violaxanthin and neoxanthin), the precursors for ABA production (Fig. 1). These results suggest that salt treatment triggers an enhanced production of β,β-xanthophylls only in roots, where their levels are about two hundred-fold lower than in the aerial part (Fig. 2A and 2B).

### ABA Production in Salt-treated Roots Depends Partially on the Supply of Carotenoid Precursors

To further investigate the relevance of the increase in β,β-xanthophyll levels for salt-induced ABA production, we carried out a similar experiment but blocked the metabolic flux into the carotenoid pathway using norflurazon (NFZ), an inhibitor of phytoene desaturation (Fig. 1). To ensure the presence of the inhibitor in roots cells during the salt treatment but prevent the undesired effects of constitutively blocking the production of carotenoids (i.e. bleaching of shoot tissues), plants grown for 2 weeks under standard conditions were transferred to fresh medium supplemented with 20 µMNFZ. After preincubation with the inhibitor for 48 h, plants were transferred to new plates containing NFZ with or without NaCl. Control plants were treated identically but transferred to media without NFZ. Root samples were collected 5 h after the last transfer to measure ABA levels. As shown in Fig. 2A, salt-triggered ABA production was substantially reduced in NFZ-treated roots, confirming that an enhanced production of the hormone partly relies on an active carotenoid pathway. Taken together, these data suggest that an enhanced supply of xanthophylls contributes to ABA production in stressed roots.

### The Increase in Carotenoid Levels in Response to Salt Stress Correlates with a Root-specific Upregulation of *PSY*gene Expression

**Figure 3:**
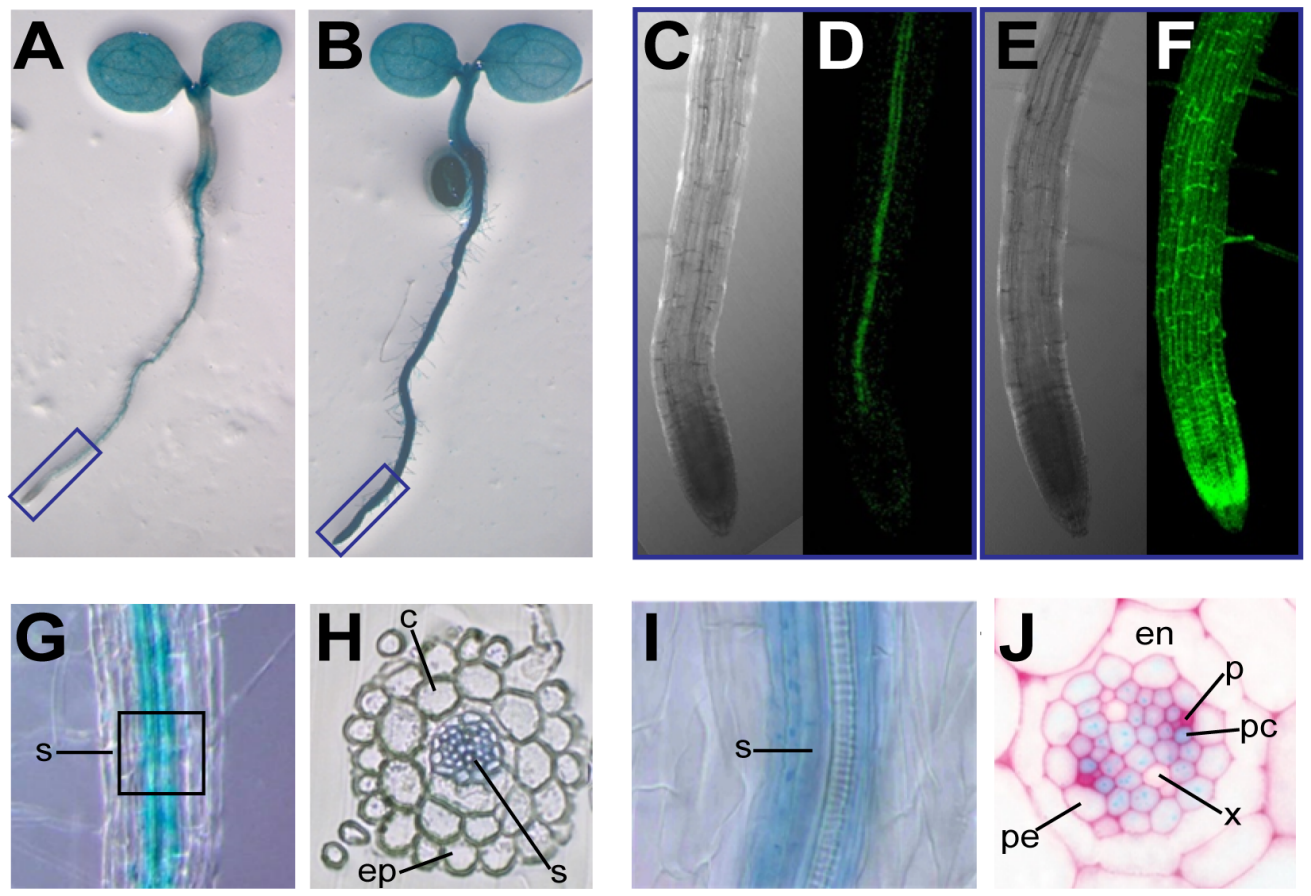
Analysis of *Arabidopsis PSY* promoter activity in roots. Representative images from transgenic *PSY:GUS-GFP* (A, C, D, G–J) and control *35S:GUS-GFP* (B, E, F) seedlings are shown. (A) GUS staining of *PSY:GUS-GFP* seedlings. (B) GUS staining of *35S:GUS-GFP* seedlings. (C) Bright field image of the region boxed in blue in A. (D) GFP fluorescence of the root shown in C. (E) Bright field image of the region boxed in blue in B. (F) GFP fluorescence of the root shown in E. (G) GUS staining of the upper region of the root shown in C. (H) Cross-section of the root region shown in G. (I) Magnification of the region boxed in G. (J) Close up of the stele area in a GUS-stained and resin-embedded section. ep, epidermis; c, cortex; s, stele; en, endodermis; pe, pericycle; p, phloem; pc, phloem companion cells; x, xylem.

The transcriptional regulation of *PSY* levels has been shown to be a major factor controlling the production of carotenoids in plants [6], [8], [15]. Remarkably, the only gene encoding PSY in Arabidopsis is expressed in most plant tissues, including roots [13], [14]. The analysis of transgenic lines harbouring a construct with the Arabidopsis *PSY* promoter fused to a reporter with beta-glucuronidase and green fluorescent protein (GUS-GFP) activities showed that this promoter was most active in the central region of the root with the only exception of the apical meristem (Fig. 3A,C,D). As expected, control lines expressing the same GUS-GFP reporter under the control of the constitutive *35S* promoter showed GUS staining and GFP fluorescence in all root tissues (Fig. 3B, E, F). A closer examination of GUS activity patterns showed that the highest levels of *PSY* promoter activity occurred in the root stele (Fig. 3G–I). In particular, the *PSY* promoter was most active in phloem companion cells and in the surrounding procambium tissues, whereas virtually no expression was detected in the xylem (Fig. 3J). Interestingly, an overlapping pattern of expression has been described for genes involved in ABA biosynthesissuch as *NCED3*, *ABA2* and *AAO3* (Fig. 1), which are highly expressed in root vascular tissues [18], [19], [20], [21]. It is therefore possible that PSY might also control the production of carotenoid precursors for ABA biosynthesis in roots. In agreement with this hypothesis, in plant species with several PSY-encoding genes (such as rice or maize) it has been described that *PSY* isoforms preferentially expressed in root tissues are upregulated upon salt stress [10], [11], [22].

**Figure 4:**
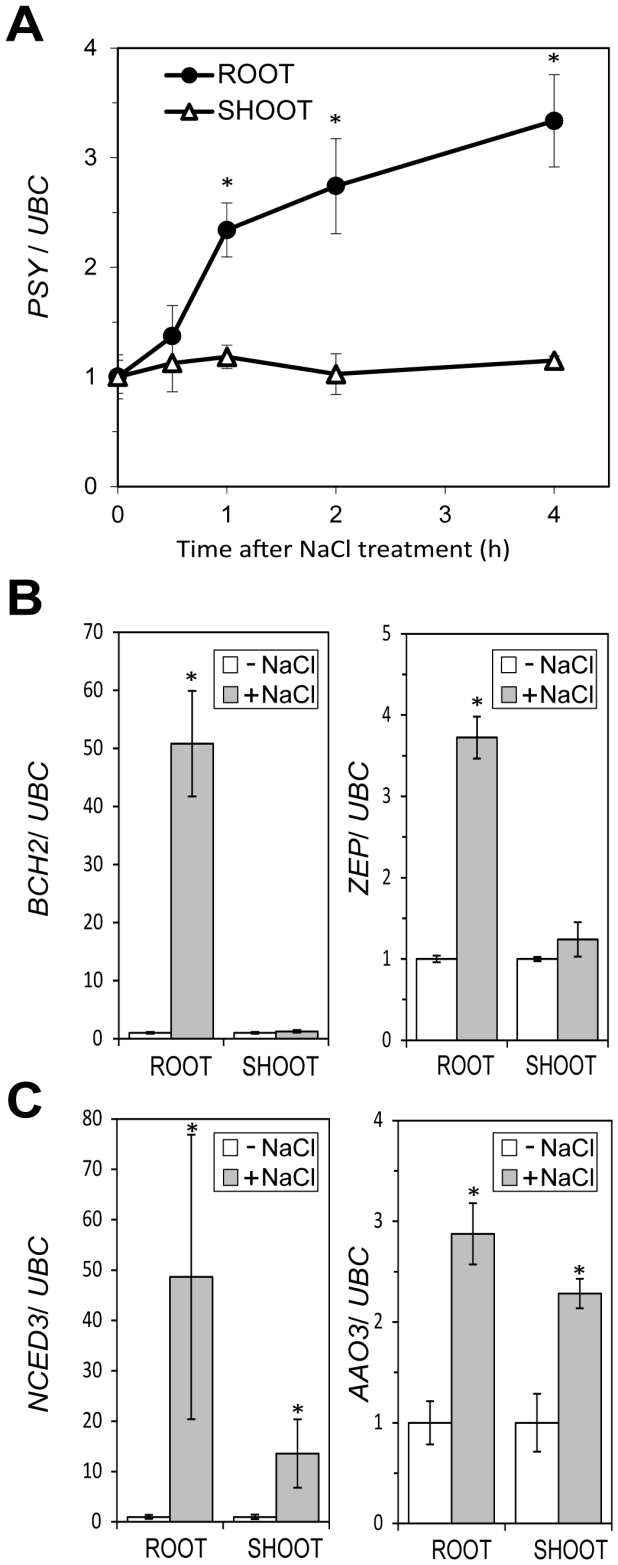
Transcript levels of carotenoid and ABA biosynthetic genes in salt-treated seedlings. After transferring plants to plates containing 200 mMNaCl for the indicated times, total RNA was separately extracted from shoot and root tissues and used for qPCR analysis of *PSY* expression (A). Transcript levels were normalized using the *UBC* gene and represented relative to those before treatment (0 h). Similarly, root samples from plants either treated (+) or not (−) with NaCl for 2 h were used to estimate the levels of transcripts from the carotenoid biosynthetic genes *BCH2* and *ZEP* (B) or ABA biosynthetic genes *NCED3* and *AAO3* (C). Data correspond to the mean and standard deviation of n = 3 (A), n = 4 (B) and n = 2 (C) independent samples. Asterisks mark statistically significant differences (p<0.01) relative to mock-treated controls.

To investigate whether the increased carotenoid accumulation observed in salt-stressed Arabidopsis roots (but not shoots) was the result of an induction of *PSY* expression, we analysed *PSY* transcript levels in root and shoot tissues that were collected separately before and after exposing plants to salinity. Quantitative real-time PCR (qPCR) experiments detected a clear upregulation of *PSY* transcript levels in roots, but not in shoots, soon after exposing plants to NaCl (Fig. 4A). This result indicates that the enhanced accumulation of carotenoids that takes place in salt-treated roots might be due, at least in part, to an increase in the metabolic flux to the carotenoid pathway supported by enhanced PSY levels.

### Other Genes Required for the Production of ABA Precursors are also Upregulated by Salt Stress in Roots

Because salinity specifically triggers an accumulation of β,β xanthophylls in roots (Fig. 2B), an induction of genes coding for enzymes that produce these compounds might also be expected (Fig. 1). In fact, a recent analysis of microarray data [23] showed that the expression of Arabidopsis genes encoding enzymes that convert β-carotene into violaxanthin such as β-carotene hydroxylases (BCH1 and BCH2) and zeaxanthin hydroxylase (ZEP) [8], [24], [25] correlates with the demand for ABA during seed development but also in response to osmotic stress. Our analysis of the data available from the Arabidopsis root eFP browser showed that NaCl increases the expression in the root stele of *PSY* but also of genes encoding β,β branchenzymes, including BCH1, BCH2, and ZEP (Fig. 1 and S1). By contrast, no changes were observed for transcripts encoding enzymes of the β,εbranch (Fig. S1).

The salt-triggered upregulation of genes encoding enzymes specific of the β,β branch was confirmed by qPCR analysis. Similar to that observed for *PSY*, the transcript levels of *BCH2* and *ZEP* were significantly higher in root samples (but not shoot samples) of salt-treated plants compared to mock-treated controls (Fig. 4B). After channelling isoprenoid precursors to the carotenoid pathway and synthesizing β,β xanthophylls, ABA is produced by the activity of enzymes like NCED3 and AAO3 (Fig. 1). A clear induction of the genes encoding NCED3 andAAO3 was observed in both aerial and root tissues (Fig. 4C). Taken together, these data indicate that, in agreement with previously reported results [1], [18], an increased ABA production in shoot (photosynthetic) tissues requires higher NCED3 and AAO3 levels but not an enhanced production of carotenoids. However, in roots, where β,β-xanthophylls are scarce (Fig. 2B), an upregulated accumulation of enzymes involved in ABA synthesis (NCED3, AAO3) but also in the supply of ABA precursors (PSY, BCH2, ZEP) might be required for sustained ABA production.

### ABA Modulates the *PSY* Upregulation Response in Roots

**Figure 5:**
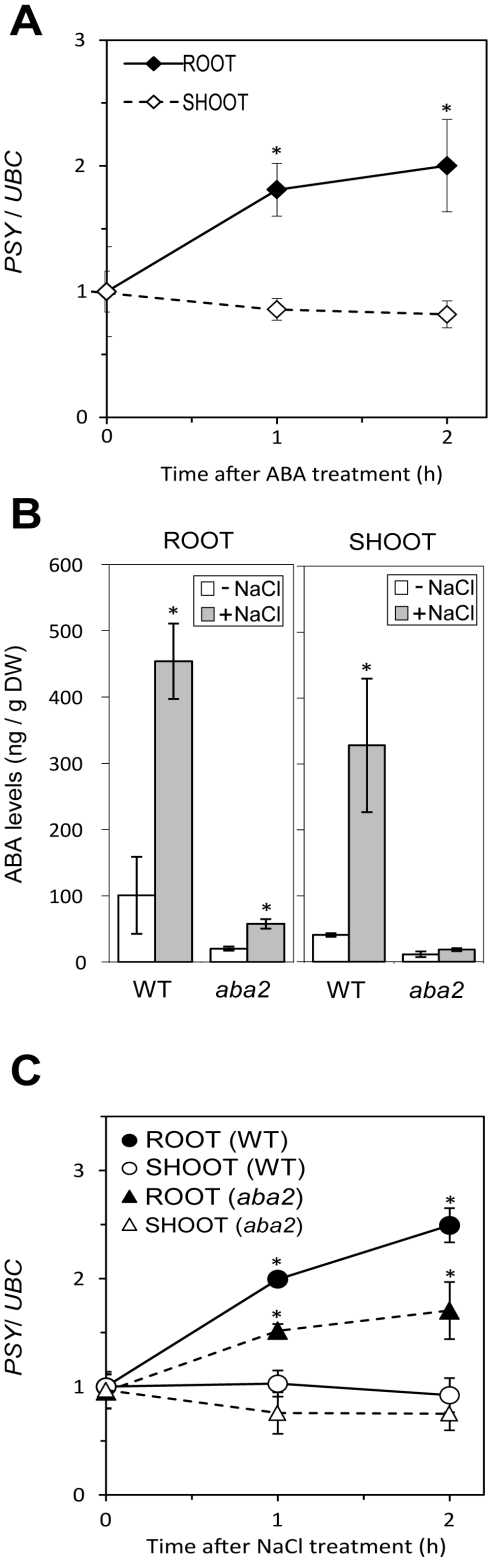
ABA effect on *PSY* expression. (A) Levels of *PSY* transcripts in root and shoot tissues from plants treated with 50 µM ABA for the indicated times. (B) ABA levels of wild-type (WT) and ABA-deficient (*aba2/gin1-3*) plants either treated (+) or not (−) with 200 mMNaCl for 5 h. Shoot and root samples were collected separately and used for ABA quantification right after salt treatment. (C) Levels of *PSY* transcripts in root and shoot tissues from WT or *aba2/gin1-3* plants after exposure to 200 mMNaCl for the indicated times. Transcript levels were normalized using the *UBC* gene and represented relative to those in samples from mock-treated plants. Data correspond to the mean and standard deviation of n = 3 (A,C) or n = 2 (B) experiments. Asterisks mark statistically significant differences (p<0.01) relative to mock-treated controls.

In previous works, the levels of *PSY* isoforms expressed in root tissues were found to be upregulated by ABA [10], [11], [22], whereas an increased expression of the Arabidopsis *PSY* gene was observed in roots after inducing ABA production by mannitol treatment [23]. To gain new insights into the possible role of ABA on the regulation of Arabidopsis *PSY* expression, we next tested whether treatment with exogenously applied ABA had any effect on transcript levels. Indeed, transferring plants to media supplemented with 50 µM ABA resulted in an increase in *PSY* transcript levels only in roots, similar to that observed in response to salt treatment (Fig. 5A). To further confirm the role of ABA on the control of *PSY* expression during abiotic stress, we compared the response to NaCl treatment in wild-type plants and mutants defective in the production of this hormone (*aba2/gin1-3*). The *aba2*/*gin1-3* mutant has a 53-bp deletion in exon 2 of the *ABA2* gene (Fig. 1) and it is considered to be a null allele [18]. Measurement of ABA levels in wild-type and *aba2*/*gin1-3* shoots and roots after transferring seedlings to standard (mock) or NaCl-supplemented medium for 5 h confirmed the presence of much lower levels of the hormone in mutant samples (Fig. 5B). Similarly, ABA is not absent but produced at very low levels and even induced after water stress in other knock-out alleles like *aba2-1* and *aba2-2* [26], [27]. As shown in Fig. 5C, the upregulation of *PSY* expression in roots of NaCl-treated *aba2*/*gin1-3* seedlings was substantially reduced relative to wild-type controls, whereas no major changes were observed in shoot tissues. All these data together confirm that ABA modulates the biosynthesis of its own metabolic precursors specifically in roots by upregulating *PSY* and, in turn, carotenoid biosynthesis, probably to cope with a higher demand of xanthophylls required for the production of the hormone.

### An ABA-dependent Mechanism to Induce Carotenogenesis in Arabidopsis Roots

Photosynthetic (shoot) tissues have high levels of carotenoids (both carotenes and xanthophylls) that contribute to photosynthesis and photoprotection. The production of ABA in these tissues mostly relies on the cleavage of available xanthophylls and therefore ABA biosynthesis is mostly regulated by the activity of NCED3 and carotenoid dioxygenases of the NCED family [3]. In agreement, our qPCR analyses (Fig. 4) showed an induction of *NCED3* but not of genes involved in the production of β,β xanthophylls (such as *PSY*, *BCH2* or *ZEP*) in shoot tissues after 2 h of transferring whole seedlings to salt-supplemented medium. Osmotic stress caused by exposing Arabidopsis roots to mannitol not only resulted in an early (and sustained) induction of *NCED3* expression but also led to a transient upregulation of *BCH2* and *ZEP* (but not *PSY*) in shoot tissues from 6 to 18 h after treatment [23]. It is therefore possible that, under some conditions, an enhanced expression of β,β branch genes might contribute to convert the abundant β-carotene intermediate into the specific xanthophylls that NCED3 cleaves to produce ABA in shoots (Fig. 1). By contrast, in non-photosynthetic tissues where carotenoid levels are low, such as roots, biosynthesis of upstream carotenoids might limit ABA production [10], [11], [23], [27], [28], [29]. The work reported here supports this model and provides evidences of a root-specific mechanism by which ABA modulates the supply of its own precursors by inducing *PSY* expression. We propose that, in response to salt stress, an increase in NCED3 and AAO3 levels (Fig. 1) results in the production of ABA from available β,β xanthophylls in both roots and shoots (Fig. 2B) [1], [18]. Additionally, sensing of abiotic stress triggers a root-specific signaling pathway that rapidly upregulates the expression of *PSY* and other genes involved in the production of β,βxanthophylls (Fig. 4), eventually providing an enhanced supply of ABA precursors for a sustained production of the hormone (Fig. 2). The restricted pattern of expression of *PSY* to the root stele (Fig. 3), similar to that described for genes transforming carotenoid precursors into ABA [18], [20], [21], suggests that most of the hormone is produced in the vasculature. ABA synthesized in the vascular tissues could rapidly be transported to other tissues to trigger appropriate responses [3].

Most plants have several PSY isoforms encoded by genes that show different expression profiles. In part due to the spatial distribution of gene expression, some isoforms are involved in the biosynthesis of carotenoids in chloroplast-containing photosynthetic tissues, whereas others participate in the production of carotenoids in the chromoplasts of the fruit, the amyloplasts of the seed, or the leucoplasts of the root [7]. The genes encoding chloroplast isoforms involved in photosynthesis are light-regulated, whereas those preferentially found in the root (such as maize and rice PSY3 enzymes) are not responsive to light but to abiotic stress and specifically to ABA [10], [11], [12], [22]. But in Arabidopsis, it exists only one single gene encoding PSY which is expressed in aerial and root tissues. In leaves, where carotenoid levels are high, *PSY* is ubiquitiously expressed, whereas in roots, where levels are low, its expression is restricted to the vascular region (Fig. 3), similar to what has been described for ABA biosynthetic genes [18], [20], [21]. This suggests that the promoter of the *PSY* gene must be able to differentially respond to signals coming from organs with distinct carotenoid requirements. It is tempting to speculate that transcription factors involved in the ABA signaling cascade might bind the *PSY* promoter preferentially in the root. Future work should additionally establish the molecular mechanisms responsible for the tissue specificity of *PSY* expression and determine the factors involved in its ABA-mediated control.

## Materials and Methods

### Plant Material and Growth Conditions

Seeds from *aba2/gin1-3* mutant and transgenic lines (*PSY::GUS-GFP* and *35S::GUS-GFP*), all in the Columbia background, were kindly provided by L.M. Lois (CRAG, Spain) or were available in the lab, respectively [15], [18]. For salt treatments, seeds were surface-sterilized and sown on a sterile mesh of filter paper or synthetic fabric (SefarNitex 03-100/44) on top of solid Murashige and Skoog (MS) medium in square culture dishes. Following stratification for 3 days at 4°C in the dark, plates were incubated vertically at 22°C under long-day (LD) photoperiod (8 h of darkness and 16 h under fluorescent white light at a photosynthetic photon flux density of 60 µmol m^−2^ s^−1^) and then the mesh with the plants was transferred to new plates containing solid MS medium either supplemented or not with 200 mM NaCl. Similarly, plants were transferred to MS plates containing 50 µM ABA for hormone treatments or 20 µM norflurazon (NFZ) prepared from Zorial (Novartis) for inhibition of phytoene desaturation. Previous experiments demonstrated the biological activity of ABA and NFZ at these concentrations in germination (ABA) and bleaching (NFZ) assays. Because the expression of the *PSY* gene oscillates during the day [30], replicate experiments were performed at the same time of the day and samples from plants treated with salt or ABA were compared to those from mock-treated controls collected at the same time.

### Histology and Microscopy

Homozygous individuals of *PSY::GUS-GFP* and control *35S::GUS-GFP* lines, were used for GUS staining and confocal microscopy of GFP fluorescence as described [15]. Roots were stained with 20 µg/ml propidium iodure (PI) to visualize cell walls as described [31]. For high resolution analysis of cell structures, GUS stained samples were embedded in Technovit 7100 (HeraeusKulzer) resin as described [32]. Sections (10 µM) were made with a Leica Jung Autocut microtome and visualized under a Leica Axiophotmicroscope.

### Analysis of Transcript Levels

After treatments, a razor blade was used to separate the roots from the shoot tissues of plants grown vertically on a mesh. RNA was then extracted from the samples and used for cDNA synthesis and qPCR analysis as described [33] using gene-specific primers for *PSY* [32], *BCH2* (G C C A A C G T T C C T T A C C T T C G and T G T G G T G T A G C T G G T G A G C G), *ZEP* (C C T T G C A T C G T C G G A A G C and G A A G GG A T C A C A A T G C G C A T), *NCED3* (C G G T G G T TT A C G A C A A G A A C A A and C A G A A G C A A T C T G G A G C A T C A A), and *AAO3* (G A G T T G G A G T C A G C G A G G T G G and T G C T C C T T C G G T C T G T C C T A A). Normalization was carried out using the *UBC-UBC21/PEX4* (At5g25760) gene [34]. Statistical differences were verified using the Simple Interactive Statistical Analysis (SISA) T-test with the default parameters (http://www.quantitativeskills.com/sisa/statistics/t-thlp.htm).

### Carotenoid and ABA Quantification

Samples were frozen in liquid nitrogen, pulverized, and lyophilized for extraction and quantification of carotenoids and ABA. Carotenoids were extracted, separated by HPLC, and quantified as described [35]. For the determination of ABA levels, extraction was carried out in ultrapure water after spiking with [^2^H_6_]-ABA as internal standard. Extracts were partitioned twice against diethylether and the organic layer was recovered and evaporated as described [36]. The dry residue was resuspended in a 90∶10 water:methanol solution and filtered prior to chromatographic separation and quantification by LC/ESI-MS/MS.

## Supporting Information

Figure S1 **Transcript levels of genes encoding carotenoid and ABA biosynthetic enzymes in the stele of Arabidopsis roots.** Data were obtained from the Arabidopsis eFP browser at www.bar.utoronto.ca [25]and correspond to stele cells collected by fluorescence-activated cell sorting of roots from 5-day-old seedlings exposed to 140 mM NaCl for 1 h [37] The position of the enzymes in the pathway is represented in the lower section of the figure.
(TIF)
