# Supplementary figures and images for: A Root Specific Induction of Carotenoid Biosynthesis Contributes to ABA Production upon Salt Stress in Arabidopsis
Source: PLoS One. 2014 Mar 4;9(3):e90765. doi: 10.1371/journal.pone.0090765 (PMC3942475; doi:10.1371/journal.pone.0090765)

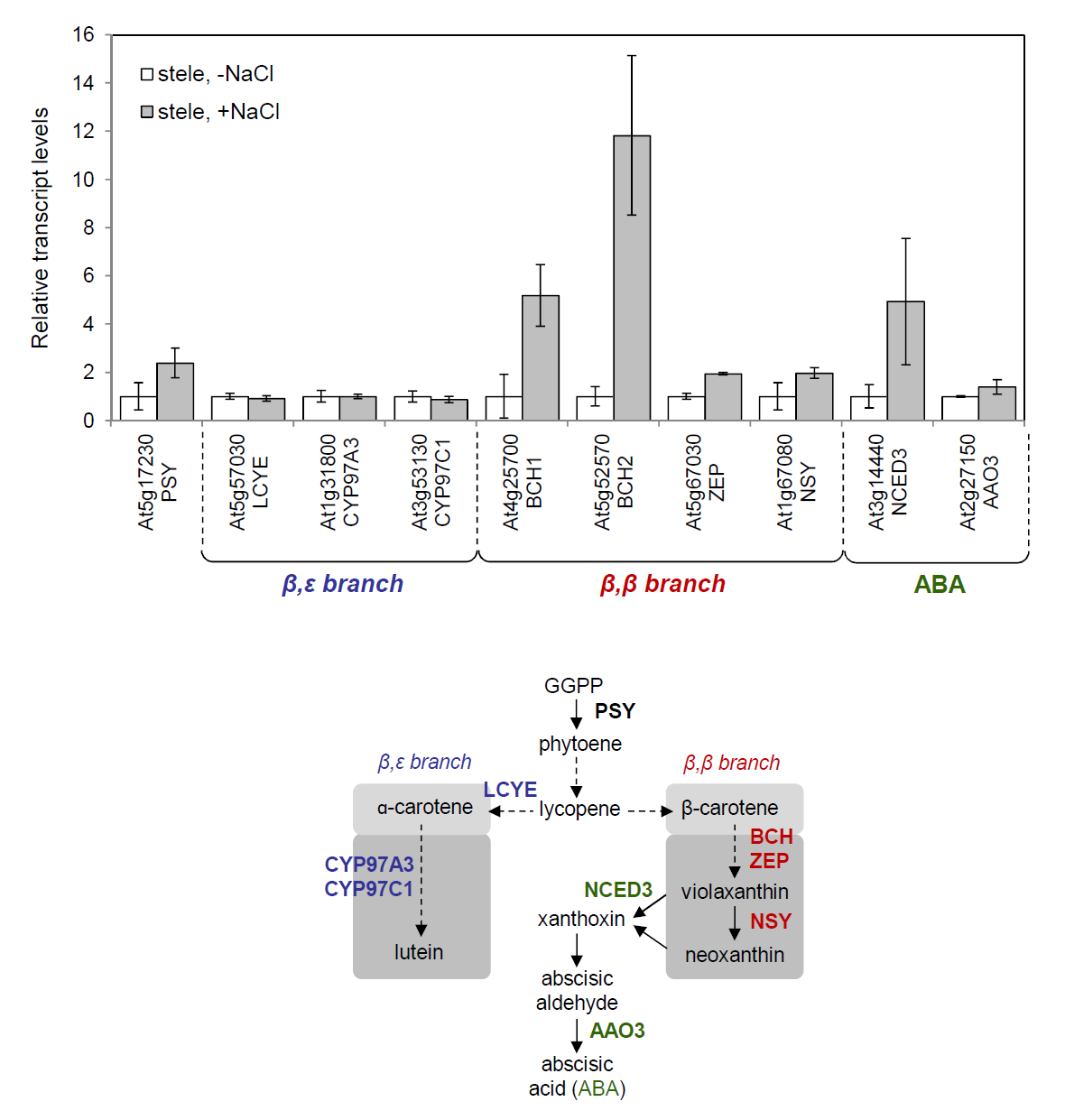

Supplement: Figure S1 — Transcript levels of genes encoding carotenoid and ABA biosynthetic enzymes in the stele of Arabidopsis roots. Data were obtained from the Arabidopsis eFP browser at www.bar.utoronto.ca [25]and correspond to stele cells collected by fluorescence-activated cell sorting of roots from 5-day-old seedlings exposed to 140 mM NaCl for 1 h [37] The position of the enzymes in the pathway is represented in the lower section of the figure. (TIF) [file pone.0090765.s001.tif]
